# Ben S. Bernanke: *21st century monetary policy: the federal reserve from the great inflation to COVID-19*: W.W. Norton & Company, 2022

**DOI:** 10.1057/s11369-022-00272-5

**Published:** 2022-08-02

**Authors:** Charles Goodhart

**Affiliations:** London, UK

Over the last four decades since the 1980s, the Federal Reserve System has been privileged to have had a series of excellent Chairs. Ben Bernanke was among the greatest of these; in addition, he is a foremost monetary economist and monetary historian. Thus, it is a great pleasure to have his thoughts on twenty-first century monetary policy, past, present, and future. I certainly learnt a lot of detail about such policies, of which I had not been aware beforehand. Everyone interested in the subject will want to read it.

The book is divided into four parts, but from my viewpoint I would divide it slightly differently. The first four chapters give his historical account of the Great Inflation and then monetary policy as managed by Paul Volcker and Alan Greenspan. Chapters 5, 6 and 7 provide an account of the conduct of monetary policy during his own spell as Chair. Chapters 8 through 10 record what has been happening to U.S. monetary policy under Janet Yellen and now Jerome (Jay) Powell. The assessment of recent developments continues through the start of the pandemic in early 2020, but with signs of a few last minute hurried additions to take account of developments towards the end of 2021, but well prior to the Russia–Ukraine war. Then, there is a long final session, about what may lie ahead, with three chapters about the future conduct of monetary policy: One chapter about financial stability and regulation, and a final short chapter on the Fed’s role in society. Most readers will be particularly interested in Bernanke’s account of events during his own Chairmanship, i.e., Chapters 5–7, and his commentary on future monetary policy, particularly Chapters 11–13.

The book is clearly aimed at a wide readership, with no use at all of math, and with all technical details either explained in simple English or simplified. It is not aimed at an academic audience. On the whole this works well, though there are a few occasions where the simplification is excessive; for example, on page 35, where it is suggested that Volcker switched to a target “for growth in the quantity of money,” avoiding any discussion of the non-borrowed reserve base. The writing is excellent, though it might have been helped by having rather more tables and charts. Bernanke has few criticisms of his predecessors or successors as Chairs; indeed, his only strong criticism of anyone is of President Trump for his attempts to bully the Fed, notably Chair Powell, in public (Chapter 9).

As noted earlier, most of us will focus primarily on the three chapters (5–7), dealing with his period as Chair. This is, of course, fascinating and a most important contribution. I did, however, have several, somewhat minor, qualifying considerations. First, I did feel that the chapters focussed mostly on his own role, serving as an apologia. The contribution of some of his Federal Reserve colleagues, notably Tim Geithner and Don Kohn, received somewhat less mention than perhaps they deserved. Second, the discussion on whether it was truly necessary to force Lehman Brothers into bankruptcy (pp. 126–127) was carefully written so as, it seems to me, to absolve himself from any criticism. Thus, the key meeting was one that he did not attend, and he then writes “after careful review of Lehman’s balance sheet, the Wall Street experts present judged it to be deeply insolvent, well beyond the point of viability unless a solvent firm acquired it.” No doubt this is absolutely correct, but given the circumstances, would not any expert from another firm have a strong incentive to assume the worst? Given the uncertainties, the experts themselves would be in great trouble should their firm acquire it and the reality turn out to be worse than the expert had predicted. Again, the worse the judgement about solvency, the more an acquiring bank could request subsidized help from the government.

Bernanke’s subsequent response, assisted by Kohn and Geithner, to deal with the resulting crisis, was one of the all time high points of central banks’ policies—flexible and innovative and successful. I had thought that quantitative easing (QE) had been a U.S. initiative, but Bernanke shows on page 148 that the Bank of England, under Mervyn King, got there first. What this clearly suggests is that the central bank’s ability to inject liquidity into the system is, in most instances, an excellent and well-working mechanism for allaying panic within the financial system.

That leads on to my main criticism of the book, which is that Bernanke focusses on QE, not just as a highly successful mechanism for dealing with liquidity crises, but rather as a general instrument for monetary expansion, even when there is no liquidity crisis at hand. I will explain why later.

Finally, Bernanke, alongside almost all central bankers of his time, has totally ignored the role and information arising from the monetary aggregates. As he points out, those monetarists in the USA who feared hyperinflation, as a result of the massive increase in the commercial bank reserve balances, caused by QE, were wrong. But this was because they had not realized that commercial banks’ demand for reserve balances had been structurally shifted by the new practice of paying interest on reserve balances. This does not mean that broader monetary aggregates had no informational value. Admittedly velocity, or demand for money, functions are often relatively unstable and can be variable over time. But so can the transmission mechanism via interest rates be unstable and variable. The intentional total disregard of monetary aggregates has been for many of us economists a mistake. When I was younger, some 50 or 60 years ago, it was common for central bankers to claim that it was extremely difficult to use monetary policy to control inflation, because of its side effects on output, unemployment, and politics, but that any central banker could easily bring about higher inflation, just by causing a faster expansion of the monetary aggregates.

Now, we have the exact opposite statement by so many central bankers, i.e., that it is extremely difficult to prevent excessive disinflation, because of the effective lower bound (ELB) on the nominal interest rate targeted by the central bank, but that central banks know how to prevent excessive inflation from getting underway, i.e., by raising interest rates, and can easily do so. But they are about to learn how difficult that is, especially now that the public sector debt ratio has been allowed to rise to such high levels.

There is hardly any mention of such rising debt ratios in both the public and private sectors in Bernanke’s book. Indeed, my main criticism of the book is that it is suffused with the implicit assumption that the future conjuncture that will face monetary policy makers will be of continuously low inflation and low nominal and real interest rates. Even by the end of 2021, the clear assumption here is that the present inflation is nothing more than a temporary bump, even though care must be taken to avoid unsettling inflationary expectations. I believe that this is wrong, and that world-wide demographic changes will restore labor bargaining power and maintain labor shortages. Even if Bernanke is right, and of course he may be, surely there is reason to fear that inflation may be more persistent from now on than he assumes. If there is any such danger that inflation might come back, possibly with a vengeance, then the continued use of QE, outside of liquidity crises, has probably been a horrible mistake, as we may soon find out. In particular, the Maturity Expansion Program (pages 174–175) had as its sole content a shortening of the outstanding duration of public sector debt. But if interest rates have been, as I believe, historically at their lowest ever point, the correct policy would have been exactly the reverse, to have locked in such extraordinarily low interest rates as far into the future as one could possibly get. Thus, outside of liquidity crises, what did we get from QE? It probably led to long-term nominal interest rates being perhaps 75 basis points lower than otherwise. Against that, we are now quite likely to see a much more difficult and dangerous time for both monetary and fiscal policies and for central bank independence. Perhaps, this shows that even the best central bankers and economists behave as ordinary people do in unduly extrapolating the recent past into the longer-term future.

The comments about future monetary policy in Chapters 11, 12 and 13, are all about the justification for QE and forward guidance against the background of lower for longer. As such, they are fine. But most of these justifications, as also for Flexible Average Inflation Targeting (FAIT) largely fall to the ground if the future could well be one of more persistent inflation. Moreover, Bernanke goes further in Chapters 12 and 13 to argue that there may need to be yet more policy tools established to deal with the dangers of potential further, and more extreme, disinflation, leading to deflation. But he never mentions what might be needed if we get much more inflationary pressure, especially now with the bloated public sector debt ratio. Nor does he ever mention, when thinking about handling deflation, mechanisms for increasing the monetary aggregates. When Bernanke looks to the future, all he sees is secular stagnation. He may be right, but then, again, he may be wrong.

When he turns to Chapter 14, on financial stability and the Fed’s role in supervision and regulation, his comments on the continuing shortcomings of U.S. macroprudential policy are well taken. In particular, it would be good if the U.S. could be in a position to vary the macroprudential controls on a counter-cyclical basis, which does not now seem possible. But even then, it could lead to a shift of financial flows to shadow banks, which are less regulated and transparent. Perhaps, a better approach would be to require CEOs of key financial institutions to be forced to return to having unlimited liability in their own company, as I have elsewhere proposed; a partial return to the practice of the early nineteenth century.

Let me conclude: This is a great book, and everyone should read it. But the mental framework and context in which it has been written just assumes that the continuing normal state of the macroeconomy will be one of disinflation and lower for longer. On that, I disagree.

It is a pity that the lag between drafting the main text for a book and its final publication is so long. Had this book been published in early 2021, or earlier, when almost everyone was still worried about inflation falling continuously below target, it would have received unstinting praise. But such has been the speed of events that its maintained assumption now looks potentially outdated.

